# Volumetric comparative analysis of anatomy through far-lateral approach: surgical space and exposed tissues

**DOI:** 10.1186/s41016-021-00268-8

**Published:** 2022-01-10

**Authors:** Ke Tang, Xu Feng, XiaodongYuan, Yang Li, XinyueChen

**Affiliations:** 1 Institute of Neurosurgery, The First Medical Center of Chinese PLA General Hospital, Fuxing Road 28, Beijing, 100853 People’s Republic of China; 2 Department of Basic Medicine, Xiamen Medical College, Guan kou zhong Road 1999, Xiamen, Fujian Province 361023 People’s Republic of China; 3 Department of Radiology, The Eighth Medical Center of Chinese PLA General Hospital, Heishanhu Road 17, Beijing, 100091 People’s Republic of China; 4 Departmentof Oral and Maxillofacial Surgery, Peking University School and Hospital of Stomatology, Zhong guan cun South Road 22, Beijing, 100081 People’s Republic of China

**Keywords:** Far-lateral approach, Three-dimensional visualization, Surgical anatomy, Quantification, Minimally invasive

## Abstract

**Background:**

The three-dimensional (3D) visualization model has ability to quantify the surgical anatomy of far-lateral approach. This study was designed to disclose the relationship between surgical space and exposed tissues in the far-lateral approach by the volumetric analysis of 3D model.

**Methods:**

The 3D skull base models were constructed using MRI and CT data of 15 patients (30 sides) with trigeminal neuralgia. Surgical corridors of the far-lateral approach were simulated by triangular pyramids to represent two surgical spaces exposing bony and neurovascular tissues. Volumetric comparison of surgical anatomy was performed using pair *t* test.

**Results:**

The morphometric results were almost the same in the two surgical spaces except the vagus nerve (CN X) exposed only in one corridor, whereas the volumetric comparison represented the statistical significant differences of surgical space and bony and neurovascular tissues involved in the two corridors (*P*<0.001). The differences of bony and neurovascular tissues failed to equal the difference of surgical space.

**Conclusions:**

For far-lateral approach, the increase of exposure for the bony and neurovascular tissues is not necessarily matched with the increase of surgical space. The volumetric comparative analysis is helpful to provide more detailed anatomical information in the surgical design.

## Background

The lesions at the inferior clivus pose a surgical challenge with high morbidity and mortality to neurosurgeons because of the critical neurovascular structures surrounding the lesion [1–4]. Many studies have described the far-lateral approach which exposed the inferior clivus superior to the anterior edge of foramen magnum [5–8]. The concern of neurovascular protection and skull base reconstruction promotes the development of surgical anatomy for the far-lateral approach [9–11].

In the previous studies, the three-dimensional (3D) visualization model had be utilized successfully in the assessment of surgical anatomy with advantage of providing quantitative details [12, 13]. The software of 3D model facilitate the volumetric measurement of anatomy [14, 15].

Researches had shown that the volumetric measurement was valuable to offer the data for the design of minimally invasive surgery [16, 17]. It is an interesting issue in the surgical design to disclose the interrelation between the surgical space and exposed tissues. We thus sought to quantify the difference of volumes for the bony and neurovascular structures in the two surgical spaces of far-lateral approach. The relationship in the anatomical exposure generated by the surgical space was demonstrated in detail.

## Methods

### Visualization of skull base

Image data of MRI and CT was acquired during Gamma Knife surgery for 15 patients with trigeminal neuralgia. The 3D visualization model of skull base was constructed in a 3D software (Mimics, Materialise US, Plymouth, Michigan). The 30 sides of skull base model were constructed for surgical simulation. The parameter of image acquisition and protocol for visualization of bony and neurovascular tissues has been reported previously [18].

### Surgical space for far-lateral approach

**Fig. 1:**
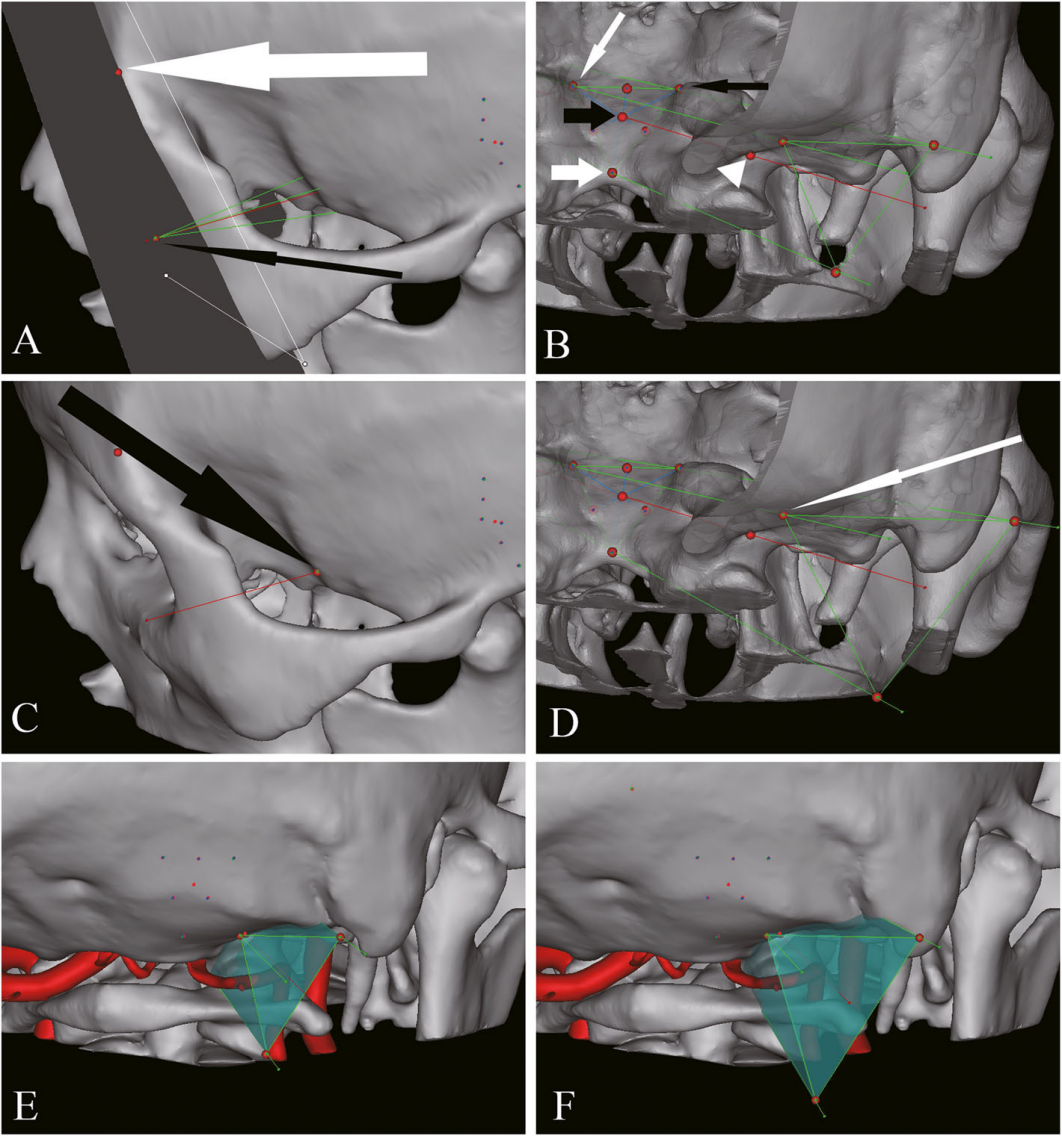
Surgical corridors outlined based on bony landmarks. **A** The top of zygomatic process of frontal bone (white thick long arrow) was used to outline plane parallel to the exposed triangle. The intersection (black thin long arrow) of the plane and the axis was the vertex of triangular pyramid for corridor 1. **B** The exposed triangle was outlined with the ipsilateral (black thin short arrow) and contralateral (white thin short arrow) anterior edge of jugular tuberculum, and anterior edge of foramen magnum (white thick short arrow) as vertexes. The axis was drawn to connect the center (black thick short arrow) of exposed triangle and the posterior and superior edge of occipital condyle (white arrow head). **C** The intersection (black thick long arrow) between axis and calvarium was the vertex of triangular pyramid for corridor 2. **D** The line on the triangular pyramid through contralateral anterior edge of jugular tuberculum form intersection (white thin long arrow) on the calvarium. Posterior view of corridor 1 (**E**) and corridor 2 (**F**)

The ipsilateral and contralateral anterior edge of jugular tuberculum and anterior edge of foramen magnum were used as vertexes to form triangle representing exposed region. We thus named this triangle as exposed triangle. The center of exposed triangle was the intersection of three midlines. The posterior and superior edge of occipital condyle was used as landmark to draw an axis connect to the center of exposed triangle (Fig. 1).

The top of zygomatic process of frontal bone was selected as landmark to outline plane parallel to the exposed triangle. The intersection of the axis on the plane was the vertex to form triangular pyramid of corridor 1 with exposed triangle as bottom. Similarly, the intersection between the axis and temporal portion of calvarium was the vertex to form triangular pyramid of corridor 2 (Fig. 1).

The line of triangular pyramid through contralateral anterior edge of jugular tuberculum crossed the occipital portion of the calvarium to form an intersection which was used to outline craniotomy plane parallel to the exposed triangle. The triangular pyramid is intersecting on the craniotomy plane to form craniotomy triangle. The space between the craniotomy and exposed triangle was defined as surgical space for both corridors 1 and 2 (Fig. 1).

### Anatomical observation and volumetric analysis

The sequence and morphology of bony and neurovascular structures in the surgical space were observed. The volume of exposed anatomical structures was calculated in the software. The volumetric comparison was performed by pair *t* test of the SPSS software (SPSS16.0 for Windows; SPSS, Chicago, IL). The *p* value less than 0.05 was used to determine statistical significance.

## Results

### Exposure sequence

The sequence of exposed anatomical tissues was almost the same in both corridors 1 and 2 of the far-lateral approach. The surgical space of simulative corridor started with bony drilling on the occipital condyle. The extra-cranial vertebral artery (VA) posterior to the occipital condyle was involved (Fig. 1).

The range of bony drilling in sequence involved the occipital condyle, occipitoatlantal joint, superior portion of lateral mass of atlas, the lateral edge of foramen magnum, posterior portion of jugular foramen and hypoglossal canal, inferior portion of jugular tuberculum, and clivus above the foramen magnum. When bony drilling arrived at the jugular foramen, the surgical space contained posterior portion of glomus jugulare and the lower end of sigmoid sinus. The surgical space also involved the intra-canal portion of hypoglossal nerve (CN XII) when crossing the hypoglossal canal.

**Fig. 2:**
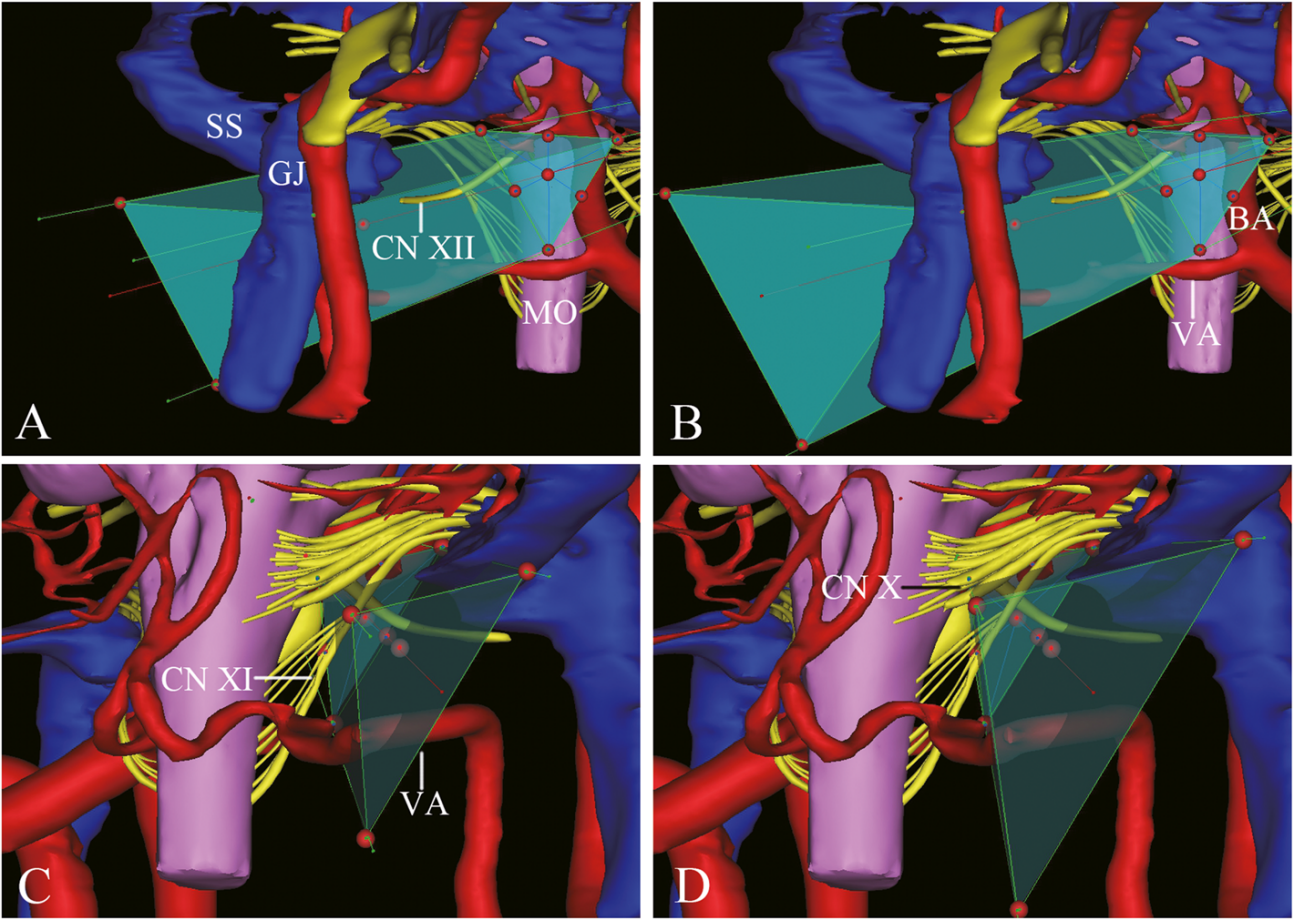
Relationship between the surgical space and neurovascular structures. **A**, **B** Anterior view of 3D reconstruction of neurovascular structures and corridors 1 and 2. **C**, **D** Posterior view of 3D reconstruction of neurovascular structures and corridors 1 and 2. BA, basilar artery; CN X, vagus nerve; CN XI, accessory nerve; CN XII, hypoglossal nerve; GJ, glomus jugulare; MO, medulla oblongata; SS, sigmoid sinus; VA, vertebral artery

For both corridors 1 and 2, the 16 sides of surgical space contained intra-cranial basilar artery (BA), whereas 14 sides did not involve BA. The surgical space passed the lateral and anterior to the cerebellum and medulla oblongata without brain tissues involved. The surgical space of corridors 1 and 2 contained the cisternal portion of accessory nerve (CN XI) and CN XII. Only the surgical space of corridor 2 involved CN X (Fig. 2).

### Volumetric analysis

The volumetric comparison represented the significant differences of anatomical exposure between corridors 1 and 2 for the far-lateral approach. The differences for all volumetric data reached statistical significance. The detail analysis was showed in Table 1, of which the volume of surgical space in corridor 2 was almost twofold of that in corridor 1. However, instead of venous structures, the increase of bony and neurovascular structure exposed in corridor 2 than 1 failed to reach twofold. Especially for CN XII exposed, the increase in corridor 2 was not obvious.

**Table 1:** Volumetric comparison of surgical anatomy

Item	Corridor 1 (mm^3^)		Corridor 2 (mm^3^)		*p* value
	Mean	SD	Mean	SD	
Operative space	5501.60	150.03	11430.00	1085.88	< 0.001
Bony drilling	1745.50	112.54	2763.50	145.15	< 0.001
Extra-cranial VA	64.05	1.34	95.53	1.08	< 0.001
BA^a^	28.90	1.84	37.86	1.41	< 0.001
Venous structures	72.36	1.63	205.69	14.10	< 0.001
CN X^b^	—	—	5.50	0.20	—
CN XI	9.61	0.40	15.38	1.21	< 0.001
CN XII	11.20	1.07	13.55	1.23	< 0.001

Comparison of the volumes (mm^3^) measured in corridors 1 and 2 (30 sides). *p* values < 0.05 indicate statistical significance. BA, basilar artery; CN X, vagus nerve; CN XI, accessory nerve; CN XII, hypoglossal nerve; VA, vertebral artery

^a^BA was involved in 16 sides of 3D model

^b^Only corridor 2 involved CN X. Thus, the pair *t* test was not performed

## Discussion

To date, the 3D visualization model becomes more popular in the study of surgical anatomy for the merits of time-saving, reproducible, and manipulation without intra-operative pressure [19–21]. Based on the 3D visualization model containing bony and neurovascular tissues, the volumetric analysis can provide detailed information in the surgical anatomy in the skull base. We thus performed this anatomical study for the far-lateral approach using the 3D model.

### Value of simulative surgical corridor

The pyramidal corridor simulating surgery can be understood as an illumination range light source of operating microscope. The vertex of pyramid is the point directed by the light source. The bottom of pyramid represents the movement range of operating microscope. The surgical space in the pyramidal corridor therefore provides an intra-operative exposure range for anatomical structures. We designed two pyramidal corridors to simulated far-lateral approach exposing clivus, of which the surgical space in corridor 2 was approximate twofold than that in corridor 1.

The 3D simulative surgical corridor offers a critical condition for volumetric analysis. The result of morphometric comparative analysis only showed that partial CN X was contained in corridor 2 instead of corridor 1, whereas the detail information of increased anatomical exposure in corridor 2 was demonstrated objectively by the volumetric analysis.

### Volumetric analysis of bony tissues

Regarding bony removal, the postoperative stabilization of the craniovertebral junction is still a concern of life quality for the patient [22]. Stability rebuilding of craniovertebral junction was required for both corridors 1 and 2 following the intra-operative damage of the occipitoatlantal joint. As the result of volumetric comparison showed, the more damage of the occipitoatlantal joint occurred in corridor 2 which would face more difficulties in the stability reconstruction.

In addition, it was time-consuming procedure to perform bony drilling [23]. Obviously, corridor 2 faced more difficulties in such manipulation for more volume of bony tissues involved. More disturbances caused by the extra-cranial VA during bony drilling also increased the difficulty of corridor 2.

### Volumetric analysis of neurovascular tissues

The surgical spaces of the two corridors exposed the cistern lateral and anterior to the cerebellum and medulla oblongata without disturbing the brain tissues, which may represent the condition of epidural manipulation to reach clivus in the far-lateral approach. When passing through the lateral edge of foramen magnum, the posterior portion of glomus jugulare and the inferior end of sigmoid sinus was involved, which has potential risk of troublesome bleeding [24, 25]. The increasing extent of venous structures in the surgical space of corridor 2 was the most.

Compared with the approximate twofold increase of surgical space, the unmatched less increase of CN XI and XII was shown in corridor 2, which implies that the difficulty of CN XI and XII protection was nearly similar in both corridors 1 and 2. Meanwhile, the CN X was only involved in corridor 2. Careful manipulation should be performed to protect the cranial nerves [26]. Due to the characteristics of position offset of the BA, only 16 sides of surgical surface involved partial BA of which the volume was slightly more in corridor 2. The mobilization of BA can be used to improve the access.

### Technical limitations

This anatomy study without real operative practice limits the clinical value. The regular geometric corridor simulating surgery is unable to complete in the current real operative conditions. We thus only demonstrate an objective comparison by volumetric analysis via regular corridors. This article highlights the relevant values of quantification for the surgical space and anatomical exposure in reference to the technical development of 3D visualization and neuronavigation.

## Conclusions

We found that the increase of surgical space exposing clivus via simulative far-lateral approach was majorly used to increase the volume of bony and venous structures. The surgical space in corridor 1 was more minimally invasive than that in corridor 2. The detailed information can be provided by the volumetric comparison.

## Data Availability

The datasets generated during and analyzed during the current study are available from the corresponding author on reasonable request.

## Abbreviations

3D: Three dimensional
BA: Basilar artery
CN X: Vagus nerve
CNXI: Accessory nerve
CN XII: Hypoglossal nerve
GJ: Glomus jugulare
MO: Medulla oblongata
SS: Sigmoid sinus
VA: Vertebral artery
